# 2D/3D ultrasound diagnosis of pediatric distal radius fractures by human readers vs artificial intelligence

**DOI:** 10.1038/s41598-023-41807-w

**Published:** 2023-09-04

**Authors:** Jessica Knight, Yuyue Zhou, Christopher Keen, Abhilash Rakkunedeth Hareendranathan, Fatima Alves-Pereira, Siyavesh Ghasseminia, Stephanie Wichuk, Alan Brilz, David Kirschner, Jacob Jaremko

**Affiliations:** 1 Department of Radiology and Diagnostic Imaging, Walter C. Mackenzie Health Sciences Centre, University of Alberta, 8440-112 Street, Edmonton, AB T6G 2B7 Canada; 2 Department of Family Medicine, University of Alberta, 5-16 University Terrace, Edmonton, AB T6G 2T4 Canada; 3 Department of Pediatrics, Edmonton Clinic Health Academy, University of Alberta, 11405-87 Avenue, Edmonton, AB T6G 1C9 Canada

**Keywords:** Trauma, Biomedical engineering, Computer science, Radiography, Three-dimensional imaging, Ultrasonography, Paediatric research

## Abstract

Wrist trauma is common in children and generally requires radiography for exclusion of fractures, subjecting children to radiation and long wait times in the emergency department. Ultrasound (US) has potential to be a safer, faster diagnostic tool. This study aimed to determine how reliably US could detect distal radius fractures in children, to contrast the accuracy of 2DUS to 3DUS, and to assess the utility of artificial intelligence for image interpretation. 127 children were scanned with 2DUS and 3DUS on the affected wrist. US scans were then read by 7 blinded human readers and an AI model. With radiographs used as the gold standard, expert human readers obtained a mean sensitivity of 0.97 and 0.98 for 2DUS and 3DUS respectively. The AI model sensitivity was 0.91 and 1.00 for 2DUS and 3DUS respectively. Study data suggests that 2DUS is comparable to 3DUS and AI diagnosis is comparable to human experts.

## Introduction

Distal radius fractures (DRF) are typically characterized as a low-energy fractures, usually due to a fall onto an outstretched hand (FOOSH), that occur approximately 2 cm proximal to the articular surface of the radius^1^. This area is particularly high risk for fractures as it is the point at which cortical bone becomes thinner and is reinforced by trabecular bone^1^. The high incidence of DRF in children can be explained by the increase in cortical porosity that results from increased bone turnover during periods of maximal longitudinal bone growth^1^. In children, males have a higher risk of DRF than females^2^. Currently, radiographs are the standard of care for diagnosis and characterization of DRF in children. Radiography of the wrist is a sensitive and clinically useful test, but it does subject children to about 1uSv of radiation per study^3^. Although 1uSv is a very small dose of radiation in comparison to average annual environmental radiation exposure (443uSv in Canada), children have a 10–15% relative risk increase of radiation induced carcinogenesis because of their increased growth rate and ongoing cellular differentiation, so care should be taken to avoid radiation exposure when possible^4,5^. Importantly, in an era of overcrowded hospitals, radiography also increases wait times in the ED. Although there is significant variety in the workflow of EDs, obtaining radiographs often requires patients and their families to move from the ED to the diagnostic imaging department. Typically, the patient must then wait for an available medical radiation technologist and await interpretation of the images before they either receive treatment for a fracture or are sent home without treatment in the absence of pathology. Fractures are usually present in only half of all children sent for radiographs with suspected DRF, meaning that half of these children could be subjected to radiation and long wait times even when no intervention is required^6^.

The use of point-of-care ultrasound (POCUS) in the ED is becoming more widespread, notably in the evaluation of patients presenting with musculoskeletal pain^7^. POCUS allows physicians to rapidly evaluate the symptomatic limb at the bedside and is quite sensitive in detecting cortical disruption, periosteal fluid, and joint effusions, all of which raise suspicion of fracture^7,8^. It has also recently been shown that physicians can detect DRF with as little as 1–2 h of hands on training^9^. As an added benefit, POCUS also allows the physician to perform dynamic assessment of muscles, tendons, and ligaments simultaneously and can easily compare the anatomy in question to the asymptomatic contralateral side^7^. US provides multiple advantages when compared to other imaging modalities which include, absence of radiation, improved patient safety, real-time image acquisition, and relatively low cost of imaging^7^. In a recent pilot study of 30 children, Zhang et al. found that 3DUS was capable of diagnosing DRF with nearly 100% sensitivity when read by a radiologist, a radiology fellow, and a medical student^8^. The findings of this pilot study are promising, however, the ultrasound (US) machines used in their study were bulky, costly and are not in wide general use which limits the clinical utility of 3DUS for DRF detection. Recently, high quality, lightweight and relatively inexpensive 2D transducers have been developed that can be used with a tablet or smartphone. These transducers have the potential to increase the utility of POCUS because they increase and are compatible with devices that most people already own^10^. They also increase accessibility to US due to their reduced cost^11^.

Even the most portable and affordable US transducer still has one key limitation, however: dedicated training with repeated exposure to both normal and abnormal anatomy is required for a user to be able to reliably and accurately acquire and interpret US images. A potential solution to this is automatic interpretation of US images with artificial intelligence (AI) which could decrease both intra-observer diagnostic variability and the training required to perform US studies for wrist injuries. Training requirements would decrease because users would only need to learn how to hold the probe and move it over the area of pain and there would be no need for training on identification of anatomy or image interpretation. The decreased need for training could increase the number of users capable of performing US scans for DRF which, coupled with the increased portability of new 2D transducers, could create new point-of-care opportunities. In the future, with a robust expanded, AI assisted protocol, it could be possible for screening for DRF or associated fractures at to be done at triage, in ambulances, or even by remote first-responders such as ski-patrol. Recent improvements in data availability and computing power have allowed deep learning models to become more widely applied in computer vision tasks, including classification, object detection, segmentation, and image synthesis. Convolutional neural networks (CNN) are a class of deep learning models that are particularly useful for imaging processing tasks^12^. In a CNN, input images are passed through several hidden layers which extract feature maps, which are then passed through output layers to generate final predictions^12^. The hidden layers involve kernels which are convolved with inputs and allow the model to extract image features at a pixel level^12^. Previous studies have demonstrated that CNN models have great potential in the field of automatic disease diagnosis and differentiation in multiple areas of medical imaging^13–15^. ResNet34^16,17^ and DenseNet121^18^ are specific types of CNN models that have unique residual blocks that reduce information loss. These models have proven to be successful in disease diagnosis related tasks such as colorectal cancer detection^19^, skin lesion analysis^20^, and pneumonia detection^21^.

Overall, we had three aims in this study: (1) to confirm the feasibility, accuracy, and reliability of US in detecting DRF in a diverse group of children, (2) to determine the human reader accuracy achievable with images from portable 2D transducers vs. traditional costly hardware with 3D transducers, and (3) to determine the feasibility of using artificial intelligence to recognize both normal wrist anatomy and fractures for the user.

## Materials and methods

### Study design

This was a prospective diagnostic study performed at the Stollery Children’s Hospital in Edmonton, Alberta. The study was approved by University of Alberta Health Research Ethics Board—Biomedical Panel (Pro00077093) and all methods were performed in accordance with the relevant guidelines and regulations.

### Study protocol

Children aged 0–17 years presenting to the Stollery Children’s Hospital ED with wrist trauma were identified at triage. Inclusion criteria were tenderness over the wrist or distal radius following trauma such as a FOOSH injury. Exclusion criteria were open fractures, lacerations in the scan area, existing cast over the scan area and the child’s inability to tolerate the exam for any reason. Written informed consent was obtained from each child’s legal guardian. Both 2D and 3D US scans were then performed in the ED waiting room immediately following triage and before the child was seen by a physician. The child was then seen separately by a blinded emergency physician for routine clinical assessment and management, of which usually included radiographs of the symptomatic wrist. All radiographs were obtained from the picture archiving and communication system (PACS) at which point children who did not receive radiographs were also excluded from the study.

2DUS and 3DUS images were randomized, anonymized and distributed to blinded volunteer readers which included 3 novice, 2 intermediate, and 2 expert readers. The corresponding radiographs were also randomized and anonymized, then centrally re-reviewed by a pediatric MSK radiologist who had been blinded to any clinical or US data associated with each patient. The radiographs were then compared to original reports done by the radiologist in the ED at the time of presentation and used as the gold standard for determining accuracy of 2D and 3D US interpretation provided by readers of novice, intermediate, and expert skill levels.

To compare accuracy of DRF diagnosis via 2DUS vs 3DUS the readers assessed each wrist data set as a whole (5 sweeps per exam), provided their diagnosis as either fractured or normal, and rated their confidence in diagnosis from -3 to + 3 (-3 = very confident no fracture; 1 = unsure but favor fracture; 3 = very confident there is a fracture).

### Training

The operators who collected the 2DUS and 3DUS scans, selected to provide a diverse range of expertise, included a medical student with 10 years of experience as a sonographer, but no prior experience in using US to screen for fractures, and an undergraduate student with no previous experience with US. Both students received a 1-h hands-on training session from a pediatric MSK radiologist and a pediatric emergency physician. Training consisted of a discussion of normal anatomy, the expected appearance of DRF, direction on how to operate both of the US machines and supervised practice performing a mock scan that followed this study’s protocol.

Novice readers were graduate students with no medical education or experience in reading or collecting US images. Intermediate readers consisted of 1 family medicine/emergency medicine physician with limited POCUS experience and the undergraduate student who helped to acquire US images for this study in the ED. The expert readers were a pediatric radiology fellow and a dual fellowship trained pediatric musculoskeletal staff radiologist with 15 years imaging experience. Novice and intermediate readers received 30 min of training which consisted of a discussion of normal anatomy of the wrist, a discussion on what constitutes an angulated and/or displaced fracture, 3 examples of a normal study and 3 examples of a DRF. The expert readers did not receive any training.

### Imaging technique

#### Ultrasound

During each US examination, the child was seated, and the affected wrist was placed in front of them on a table in a neutral position. 3DUS images of the symptomatic wrist were then acquired with a Philips IU22 machine using a 13 MHz VL13-5 probe (Philips, Amsterdam, NL). Next, 2DUS images of the same wrist were acquired with a Philips Lumify L5-12 MHz probe (Philips, Amsterdam, NL) and a tablet computer using Android OS (Alphabet Inc, Mountain View, CA).

**Figure 1:**
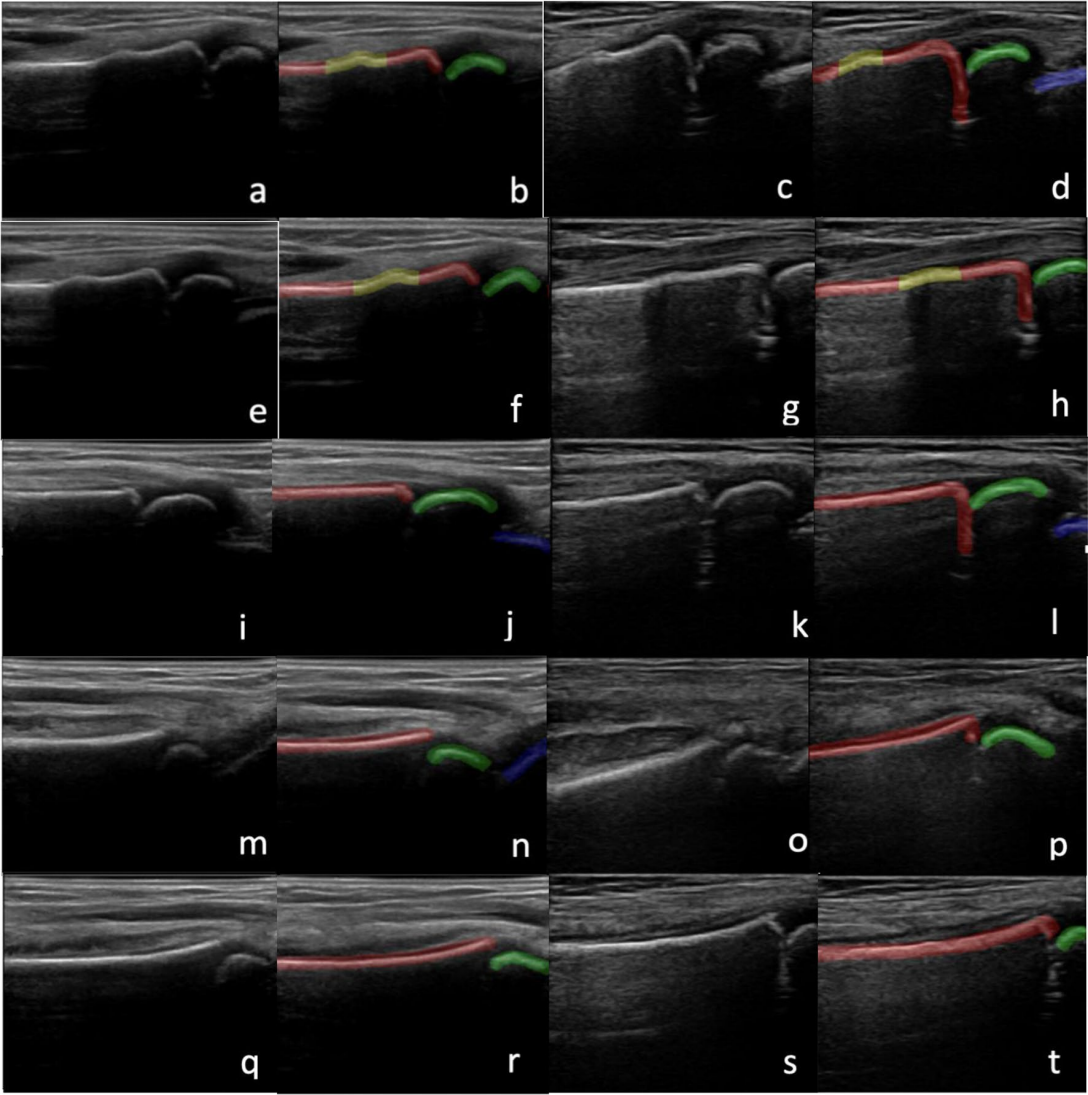
Images with corresponding manual labels of DRF including 3D dorsal (**a,b**), 2D dorsal (**c,d**), 3D proximal dorsal (**e,f**), 2D proximal dorsal (**g,h**), 3D radial (**i,j**) 2D radial (**k,l**), 3D volar (**m,n**), 2D volar (**o,p**), 3D proximal volar (**q,r**) and 2D proximal volar (**s,t**) views. Labels include radial metaphysis (red), epiphysis (green), carpal bones (blue) and fracture (yellow).

Images acquired with each machine included (1) the dorsal aspect of the distal radius with metaphysis, epiphysis, and first row of carpal bones visible, (2) a more proximal portion of the dorsal aspect of the distal radius with metaphysis and epiphysis visible, (3) the radial aspect of the distal radius with metaphysis, epiphysis, and first row of carpal bones visible, (4) the volar aspect of the distal radius with metaphysis, epiphysis, and first row of carpal bones visible and (5) a more proximal portion of the volar aspect of the distal radius with metaphysis and epiphysis visible (Fig. 1). Upon completion of this imaging protocol we were left with 5 3DUS sweeps and 5 2DUS sweeps for a total of 10 US sweeps for each symptomatic wrist.

#### Radiographs

Each child included in this study also had radiographs of the symptomatic wrist and/or forearm obtained as part of routine clinical care at the same ER visit. We recorded the diagnosis (DRF vs no DRF) for these images based on the initial radiologist report at the time of presentation, then compared the initial diagnoses to those produced by a blinded re-read of all radiographs done by our expert pediatric MSK radiologist. We also reviewed patient charts for a 30-day period following the ER visit to determine whether any had an initially occult fracture that was detected on follow-up imaging. Diagnosis from our blinded re-review of radiographs was then treated as the gold-standard for this study as it limited intra-observer variability and was blinded to clinical data unlike the initial radiologist reports.

### Artificial intelligence

#### Image labeling

An experienced sonographer manually labeled each US image that was contained within the US sweeps (Fig. 1). Labeling was done using ITK-Snap (version 3.8.0) and included radial metaphysis (red), radial epiphysis (green), carpal bones (blue) and fractures (yellow). Images with a yellow label were categorized as positive for fracture, and those without a yellow label were categorized as negative. Individual fracture labels were compared with gold standard radiographs. Only individuals with the same label generated by manual segmentation and radiographs were used for AI model training and evaluation.

#### AI training

Each image was cropped to contain only the US data. Only images that contained the radial metaphysis were used for model training, validation and testing. The dataset was split randomly into training, validation and tests set based on anonymized study ID. As most study IDs had 2D and 3D data, each patient's train/validation/test set was kept consistent between 2 and 3D datasets to avoid data leakage (Table 1).

**Table 1:** 2DUS image distribution between training, validation and tests sets.

	2D training	2D validation	2D test	3D training	3D validation	3D test
Number of images (% fractured)	16,865 (24.81%)	4215 (20.90%)	3822 (22.42%)	15,882 (32.33%)	3787 (33.25%)	4034 (28.04%)
Number of patients (% fractured)	76 (56.58%)	17 (58.82%)	18 (61.11%)	71 (57.75%)	15 (66.67%)	18 (61.11%)
Number of Sweeps (% fractured)	370 (33.24%)	84 (27.38%)	89 (31.46%)	349 (39.54%)	75 (41.33%)	89 (39.33%)

Before being fed into the model, images were first processed by zero-padding to squares and then resized to 364*364 and normalized. Stochastic gradient descent (SGD) was used as the optimizer, with a learning rate of 0.002 and momentum of 0.9 for all of the models. Cross-entropy loss was used as the loss function. We did experiments on: hyperparameter tuning on optimizer weight decay, data augmentation with random horizontal flip, training set data distribution rebalance using Imbalanced Data Sampler. ResNet34 and DenseNet121 were trained with default model architecture for fracture detection based on single US images. The final fully connected layer output was set to be 2 for binary classification. Softmax was used to scale the prediction between 0 and 1. All the models were trained for 100 epochs with 16 as batch size. Models were trained on Compute Canada using a V100 GPU. Training details of each model with the highest validation AUROC can be found in Table 2.

**Table 2:** Training details of models with highest validation AUROC with 2DUS and 3DUS.

	ResNet34 with 2DUS	Densenet121 with 2DUS	ResNet34 with 3DUS	Densenet121 with 3DUS
SGD optimizer weight decay	0.001	0.001	0.005	0.001
Random horizontal flip	No	No	Yes	No
Training data rebalance with imbalanced data sampler	Yes	Yes	No	Yes

### Statistical analysis

Sensitivity (SN), specificity (SP), positive predictive value (PPV), negative predictive value (NPV), positive likelihood ratio (LR +) and negative likelihood radio (LR-) were calculated for human readers and AI using our gold-standard radiographs for diagnosis of DRF. Accuracy and area under the receiver operating characteristic curve (AUROC) were also calculated for AI. The model with the highest AUROC on the validation set was then used on the test set. Interrater reliability was calculated via, Fleiss’ Kappa and Cohen’s Kappa using Microsoft Excel^22–24^. Differences between human US interpretation and gold standard radiograph re-reviews were evaluated for statistical significance with McNemar's test using Microsoft Excel^25^.

### Informed consent

Written informed consent was obtained from all subjects and legal guardians involved in this study.

## Results

### Clinical data

This study enrolled 127 children, with 122 children receiving both US and radiographs, resulting in 1165 individual US sweeps of symptomatic wrists. Each sweep produced a DICOM file composed of a minimum of 90 individual sequential US images. Due to technical issues, time constraints or a child’s request to stop the examination 8 children received a 2DUS scan only and 3 children received a 3DUS scan only. The 5 children who did not receive radiographs and therefore could not be compared to the gold standard were excluded from the study.

**Figure 2:**
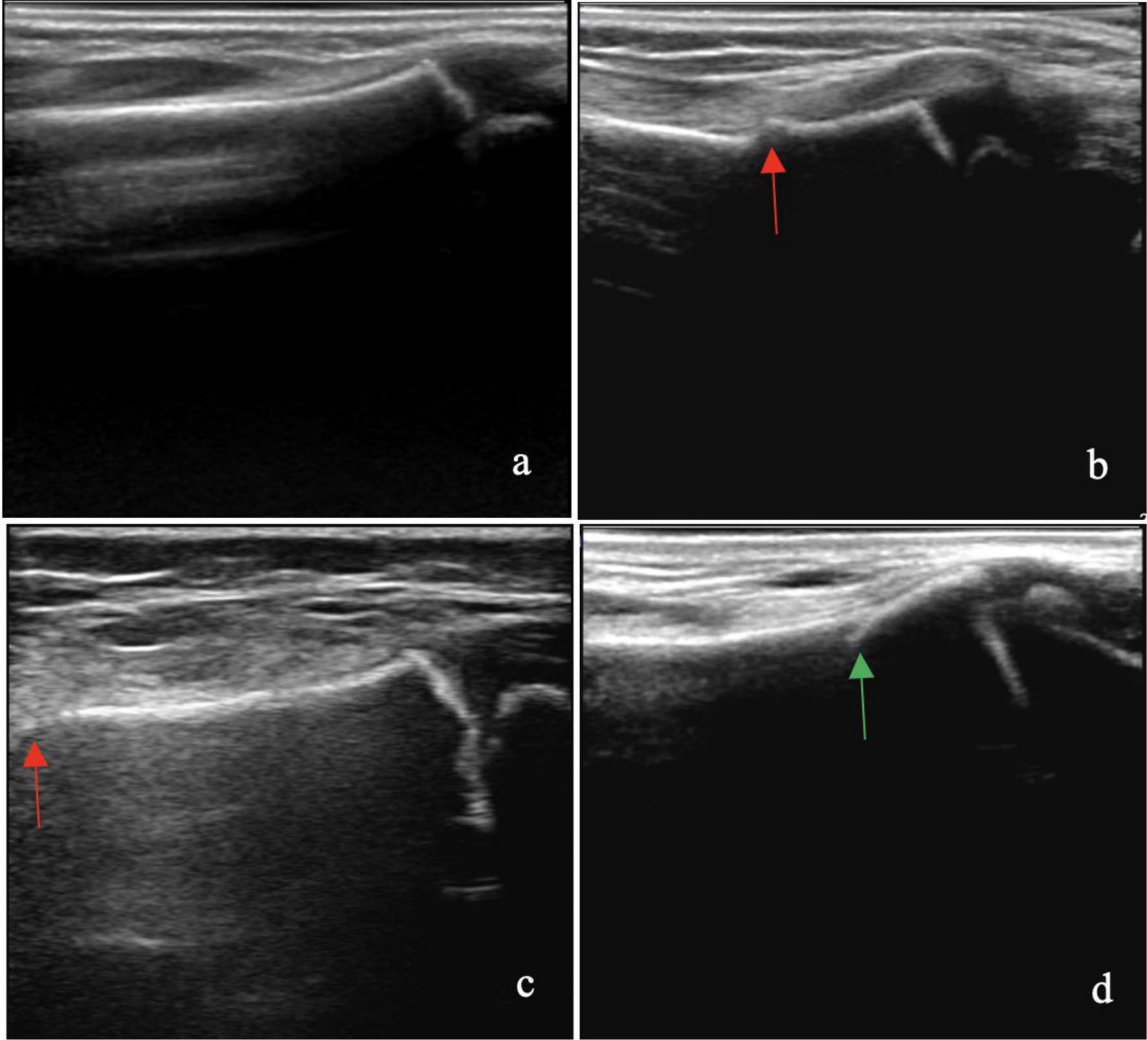
Examples of clearly normal (**a**) and clearly fractured (**b**) distal radius and examples of a subtle, but detectable fracture near the edge of the field of view (**c**) and an artifact (**d**). Fractures are labeled with a red arrow and the artifact is labelled with a green arrow.

Blinded, asynchronous novice, intermediate and expert reader impressions of 2D and 3D US images showed improving SN with increasing experience of the reader when impressions were compared to the initial radiologist radiograph reports (Tables 3 and 4). Since the radiographs collected in this study were read by many different radiologists depending on the date and time of presentation, a blinded central re-review of all radiographs was done by a pediatric MSK radiologist in an effort to eliminate inter-observer variability. This interpretation was used as our gold standard classification. The reader impressions from 2 and 3DUS were compared to our gold standard radiograph interpretations and again showed improving SN with increasing experience of the reader (Tables 3 and 4). Discrepancies between initial radiologist report diagnosis and blinded re-review diagnosis were minimal, with all discrepant scans having subtle or questionable findings for which there would be expected disagreement between readers (Fig. 2, Table 5). Inter-rater reliability was fair to moderate overall, but very good between experts (Table 6).

**Table 3:** 3DUS DRF detection by novice, intermediate and expert human readers when compared to gold standard re-reviewed radiographs.

3D	Novice value (range) *(p* = < *0.0001)*	Intermediate value (range) *(p* = *0.044)*	Expert value (range) *(p* = *0.182)*
Sensitivity (gold standard)	0.63 (0.32–0.84)	0.88 (0.78–0.98)	0.98 (0.98–0.98)
Specificity (gold standard)	0.91 (0.88–0.96)	0.95 (0.93–0.96)	0.93 (0.92–0.94)
PPV (gold standard)	0.90 (0.89–0.92)	0.96 (0.96–0.96)	0.95 (0.95–0.96)
NPV (gold standard)	0.67 (0.51–0.81)	0.87 (0.77–0.98)	0.97 (0.96–0.98)
+ LR (gold standard)	7.05 (5.75–8.10)	17.25 (15.75–18.75)	13.78 (11.81–15.75)
− LR (gold standard)	0.40 (0.17–0.71)	0.13 (0.02–0.23)	0.02 (0.02–0.02)

**Table 4:** 2DUS DRF detection by novice, intermediate and expert human readers when compared to gold standard re-reviewed radiographs.

2D	Novice value (range) *(p* = < *0.0001)*	Intermediate value (range) *(p* = *0.055)*	Expert value (range) *(p* = *0.181)*
Sensitivity (gold standard)	0.62 (0.41–0.80)	0.88 (0.80–0.95)	0.97 (0.95–0.99)
Specificity (gold standard)	0.82 (0.69–0.90)	0.94 (0.90–0.98)	0.90 (0.90–0.90)
PPV (gold standard)	0.82 (0.74–0.91)	0.95 (0.91–0.98)	0.93 (0.93–0.93)
NPV (gold standard)	0.64 (0.54–0.77)	0.86 (0.77–0.94)	0.96 (0.94–0.98)
+ LR (gold standard)	4.41 (2.11–7.84)	27.29 (7.84–46.74)	9.50 (9.35–9.65)
− LR (gold standard)	0.47 (0.22–0.68)	0.14 (0.05–0.22)	0.04 (0.02–0.05)

**Table 5:** Discrepancies between initial radiologist radiograph report and gold standard re-reviewed radiograph diagnosis.

Study ID	Initial	Gold standard	Gold standard re-review comment
29	Normal	Fractured	Very subtle and quite distal
38	Fractured	Normal	Suspicion of non-displaced salter 2
130	Normal	Fractured	Quite distal
160	Normal	Fractured	Slight ripple volar only
165	Normal	Fractured	Slight ripple volar only

**Table 6:** Inter-rater variability using Cohen’s and Fleiss’ Kappa for 2DUS, 3DUS and 2D/3DUS combined.

Reliability (kappa)	2DUS	3DUS	Overall
All 7 readers	0.48	0.55	0.30
Novice readers	0.15	0.31	0.24
Intermediate readers	0.72	0.69	0.70
Expert readers	0.86	0.91	0.84

**Figure 3:**
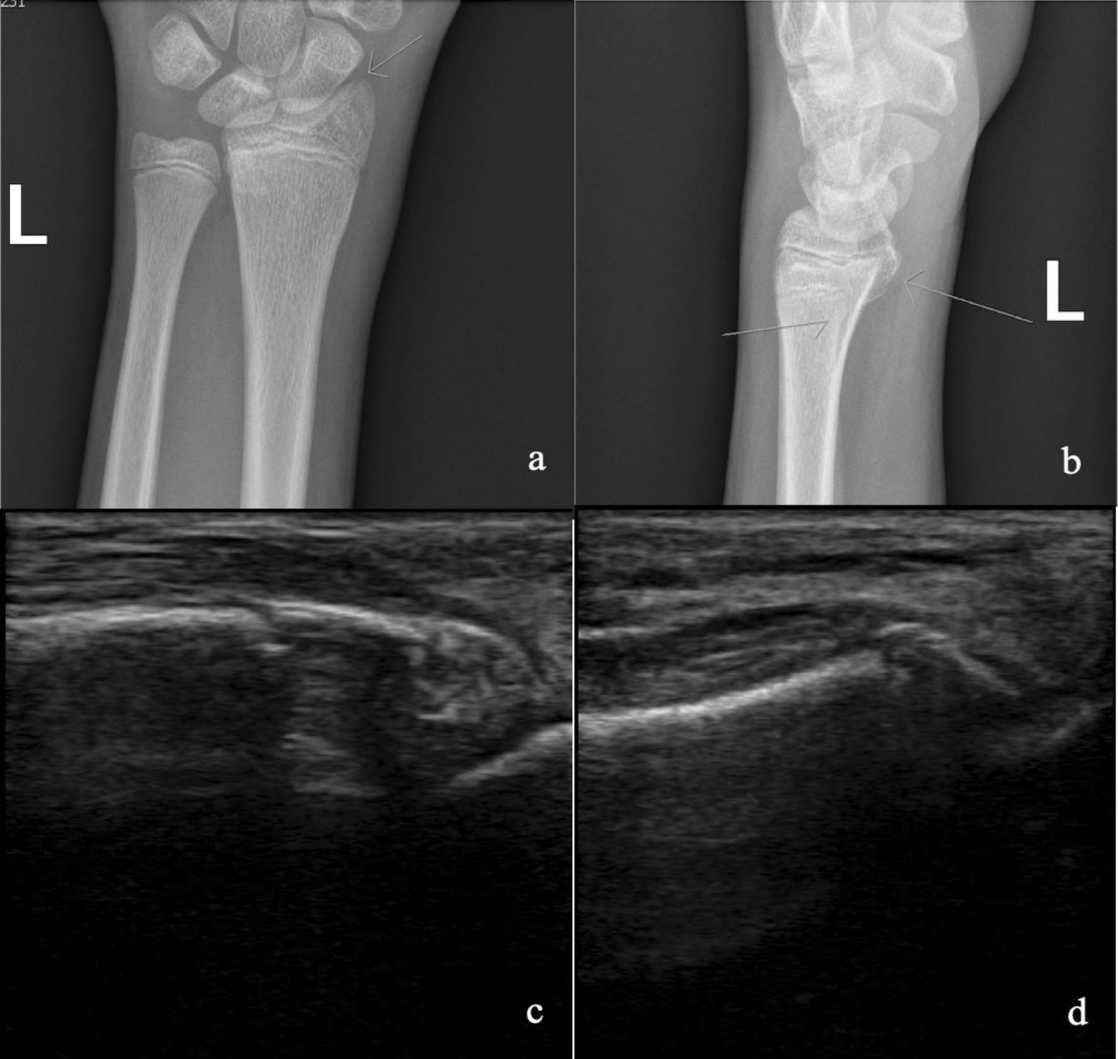
AP (**a**) and lateral (**b**) radiographs of a false negative US case, found to be a near anatomical, non-displaced Salter Harris 2 fracture, compared to radial (**c**) and volar (**d**) views of the corresponding area on 2D US. In hindsight there is a disruption in the physis cortex visible on 2DUS, though this was not appreciated at the time of the blinded expert read. This highlights the difficulty identifying a fracture when it is non-displaced and involves an open physis, which is already a discontinuity in cortex.

Once the blinded multi-reader trial was complete, all gold standard radiograph findings and their corresponding US images were reviewed for discrepancies. It was noted that multiple patients had upper extremity fractures that were seen on the radiographs but were outside the distal portion of the radius that was assessed with US. There were 38 patients with fractures elsewhere in their upper extremity, with 35 of these patients having ulna fractures and 3 patients with radial diaphysis fractures that were proximal to the scan area. There was also one wrist that was read by both experts as normal on ultrasound but was in fact fractured. This false negative wrist was a near anatomical, non-displaced Salter Harris 2 fracture and was the only fracture involving the physis found in our study data (Fig. 3).

### Artificial intelligence data

#### Single image fracture detection

The first step in testing the models’ classification capability was to test them on single images. The threshold for fracture prediction was set to 0.5, which meant that if the fracture prediction probability was equal to or higher than 0.5 it was classified as positive for DRF and was otherwise negative. The model performed well with both ResNet34 and Densenet121 achieving an AUROC of 0.93 and 0.91 for 2DUS and 3DUS respectively.

#### Patient level fracture detection

Once it was determined that the models could classify single images as positive or negative for DRF, we evaluated their performance on the patient level, where the classification of all images belonging to the same patient were used to come to a conclusion about the patient’s diagnosis of either having a DRF or not. For this process 2D and 3D images were kept separate and treated as a separate study.

For 3DUS, patients were classified as having a DRF if there were 15 or more consecutive single image positive predictions by the model. For 2DUS patients were classified as having a DRF if there were 11 or more consecutive single image positive predictions. The thresholds for consecutive positive predictions were determined based on the validation set ground truth label distribution. For each patient, we counted the number of images with fracture for each video and found the smallest number among those videos. We set 0.5 (the smallest number) as the threshold to determine if there was a positive prediction on video level. Any individual with at least 1 positive video prediction was considered a patient with positive prediction. Densenet121 was the most sensitive network for detecting DRF in both 2DUS and 3DUS (Table 7).

**Table 7:** ResNet34 and DenseNet121 model performance on patients as a whole using 2DUS and 3DUS images.

	ResNet34 with 2DUS	Densenet121 with 2DUS	Human Expert with 2DUS	ResNet34 with 3DUS	Densenet121 with 3DUS
Accuracy	0.89	0.94		1.00	0.94
SN	0.82	0.91	0.89	1.00	1.00
SP	1.00	1.00	0.85	1.00	0.86
PPV	1.00	1.00	0.90	1.00	0.92
NPV	0.78	0.88	0.83	1.00	1.00
LR +	Infinite	Infinite	5.82	Infinite	7.00
LR −	0.18	0.09	0.14	0.00	0.00

## Discussion

The data from this study suggests that US is already an accurate and reliable tool for DRF diagnosis in the hands of experienced readers and that it is feasible to perform US on children presenting to the ED with wrist injuries as early as at triage. The data also agrees with our hypothesis that 2DUS and 3DUS image quality were comparable and they can both be used by expert human readers to detect DRF with sensitivity and specificity as high as 97% and 98% respectively. The AI networks used in this study also demonstrate that AI can interpret US images with accuracy similar to human experts, producing sensitivities of 91% and 100% for 2DUS and 3DUS respectively. While interpretation of US images by novice and intermediate readers showed overall moderate agreement with expert interpretation, the interobserver variability within these two groups puts the reliability of an inexperienced user’s diagnosis into question. The variability between readers further highlights the utility that our AI model could have in increasing the accuracy and reproducibility of DRF US diagnosis in the hands of inexperienced users.

Since 2DUS has been found to be comparable to 3DUS in fracture detection by human readers, the issues of cost and portability that would likely impede clinical use of US for DRF diagnosis have been diminished significantly. To further improve the clinical utility of using US for DRF detection, next steps could include app development for automated AI interpretation of 2D images on smartphones and tablets for use with low cost, portable transducers. However, more work needs to be done in order to create an AI network that is capable of detecting DRF with 100% SN using 2DUS, as we have done with 3DUS in this study. The use of app-based AI US interpretation would decrease the need for extensive in-person training US which is one of the most important limiting factors for the use of POCUS of any kind^26^. Increased accessibility coupled with automatic, real-time AI interpretation of US images has the potential to make US for DRF detection a valuable and readily available decision-making tool for clinicians, and even for first responders or healthcare professionals in remote areas. Importantly, if used at triage as was done in this study, AI assisted US for DRF could identify wrists that are normal with high confidence and prevent children without fractures from having to wait in the ED at all. Decreasing the number of children without DRF requiring radiographs or physician assessment could improve timely access to treatment for the children who do have DRF. In a recent study by Korup et al. it was estimated that children aged 0–17 years suffer DRF at a rate of approximately 738.1 per100,000 every year, which means using US for DRF diagnosis has the potential to decrease ED wait times for approximately 630,000 children every year in North America alone^27–30^. Decreasing the number of children sitting unnecessarily in ED waiting rooms would also help to address problems with over-crowding and would allow for improved physical distancing and isolation of sick patients also awaiting treatment.

There were limitations to our study. Although we compiled data from 122 children, this was still a single-institution trial and results should be confirmed in a future multicenter study. Statistical power could be increased by recruiting more readers of novice, intermediate and expert skill level. Recruiting readers was difficult because assessment of images was time-consuming and secure transfer of large data sets was cumbersome. In addition, radiographs were used as our gold standard and they are only 95% sensitive in detecting radius fractures when compared to CT^31^. We were unable to use more sensitive modalities like MRI and CT for logistical and ethical reasons. We also found that, while expert US readers had 100% sensitivity in detecting all metaphyseal fractures (displaced and non-displaced), they can miss non-displaced physis fractures. Since there was only one non-displaced physis fracture included in our study, further investigation is required to determine the true detection rate of these more subtle fractures. Lastly, as with most AI networks, ours are not fully explainable. However, our model does not just generate a binary classification of 'fractured' vs ‘normal’, it also provides a segmentation mask, i.e., a color-coded model showing the user the labelled bones and directly highlighting any fractures. This model output improves the explainability of the AI and could help increase uptake and trust among clinicians compared to a pure classification network.

The presence of 38 forearm fractures not involving the distal radius in our patient group suggests that screening the entire forearm in children presenting with upper extremity trauma should be investigated in future studies to reduce false negatives due to non-imaged pathology. Now that we have created a model that can accurately detect DRF, with adjustments to the ultrasound scan protocol and investigation into the sensitivity of US for detecting other upper extremity fractures, the AI tool could be extended to detect DRF in adults, radial diaphyseal fractures, ulnar fractures and potentially even scaphoid fractures. An additional area of future exploration would be to determine whether or not AI vs novice, intermediate or expert users can accurately classify a DRF as displaced or angulated. Information about displacement and angulation could further increase clinical utility of US diagnosis as these are valuable parameters in determining if reduction will be required for treatment.

## Conclusions

We found that both 3DUS and portable 2DUS are reliable tools for diagnosing pediatric DRF when compared to radiographs. Accuracy and reproducibility of US diagnosis increases with reader experience with experts achieving sensitivities as high as 98%. AI diagnosis with our model is comparable to that of expert human readers. Real-time app-based automated AI diagnosis of 2DUS images has the potential to increase accessibility and reliability of US for DRF diagnosis in the hands of inexperienced users and could help to decrease ED wait times if used upon triage.

## Data Availability

The data presented in this study are available upon request from the corresponding author. The data are not publicly available due to patient privacy requirements of clinical data.
